# Reduction in Synaptic Vesicle Protein Abundance but Increased Amounts of Nsg2 and Lpcat1 in Cerebral Cortices Without the Endosomal SNARE Proteins Vti1a and Vti1b

**DOI:** 10.1002/pmic.70117

**Published:** 2026-03-20

**Authors:** Julia Gottschalk, Katharina Kotschnew, Julia Hahn, Thomas Patschkowski, Gabriele von Fischer von Mollard

**Affiliations:** ^1^ Biochemistry III Department of Chemistry Bielefeld University Bielefeld Germany; ^2^ Proteom and Metabolom Research Department of Biology Bielefeld University Bielefeld Germany; ^3^ Technology Platform Genomics CeBiTec Bielefeld University Bielefeld Germany

**Keywords:** endosome, membrane traffic, neurodegeneration, proteome, SNARE

## Abstract

Absence of the endosomal SNAREs vti1a and vti1b results in perinatal death and severe neuronal phenotypes in mice, while lack of one of these proteins results in minor phenotypes. Proteomic differences were investigated to obtain a deeper insight into processes in which vti1a and vti1b are involved. Here we applied a bottom‐up shotgun proteomic approach to investigate the differences in wild‐type, double heterozygous (DHET), vti1a*^−/−^* vti1b*^−/−^* double knockout (DKO), vti1a*^−/−^* knockout and vti1b*^−/−^* knockout cerebral cortices. Single deletions did not affect protein levels significantly. A total of 1725 proteins were detected of which 69 were less abundant and 191 proteins were more abundant in DKO cortices. Many less abundant proteins belonged to cellular components and reactome pathways synapse, synaptic vesicle cycle, vesicle mediated transport, L1CAM interaction, and cholesterol biosynthesis in pathway enrichment analysis. More abundant proteins were enriched in cellular components and Kyoto Encyclopaedia of Genes and Genomes (KEGG)‐pathways such as spliceosome, ribosome, carbon metabolism, and ribonucleoprotein complex. Immunoblotting validated reduced expression levels of the tested synaptic vesicle proteins as well as increased amounts of lysophosphatidylcholine acyltransferase 1 (Lpcat1) and neuron‐specific gene 2 (Nsg2), which is involved in postsynaptic AMPA‐receptor recycling. These data indicate that the synapse and cell adhesion were strongly affected in DKO brains.

**Statement of Significance of the Study:**

Distinct populations of neurons and glia cells are generated and organize into layers during brain development. Neurons develop an elaborate morphology to transmit information via axons and synapses to dendrites in receiving neurons. These neurites form via several specialized pathways of vesicle secretion and endocytosis. Fusion between these membranes requires members of the SNARE protein family. Double knockouts of the endosomal SNAREs vti1a and vti1b (DKO) result in perinatal lethality in mice with massive defects in the brain. In this study we compared the proteome of DKO brain cortices with double heterozygous controls to obtain insights into the molecular alterations and affected pathways. DKO brains contained lower amounts of synaptic proteins and proteins involved in cell adhesion, membrane trafficking and cholesterol biosynthesis. Several proteins of spliceosomes, ribosomes and carbon metabolism were more abundant in DKO brains, which may be a consequence of the reduced amounts of synaptic proteins or a shift in cell populations. Lysophosphatidylcholine acyltransferase 1 (Lpcat1) and neuron‐specific gene 2 (Nsg2), which is involved in α‐amino‐3‐hydroxy‐5‐methyl‐4‐isoxazolepropionic acid receptor (AMPAR) recycling, were confirmed to be more abundant by Western blotting. These data point to defects in trafficking especially in the synapse and in cell adhesion, which is required for neurite outgrowth.

**Abbreviations**
Ahsg: fetuin
Alb: albumin
AMPAR: α‐amino‐3‐hydroxy‐5‐methyl‐4‐isoxazolepropionic acid receptor
Ank2: ankyrin‐2
BDNF: brain‐derived neurotrophic factor
DHET, DH: vti1a*^+/−^* vti1b*^+/−^* double heterozygot
DKO: vti1a*^−/−^* vti1b*^−/−^* double knockout
FcRn: neonatal Fc receptor for IgG
FDR: false discovery rate
GO: gene ontology
KEGG: Kyoto Encyclopaedia of Genes and Genomes
Lpcat1: lysophosphatidylcholine acyltransferase 1
Nsg2: neuron‐specific gene 2
PCA: principal component analysis
Serpin1b: α1‐antitrypsin 1–2
SNARE: soluble *N*‐ethylmaleimide‐sensitive‐factor‐attachment receptor
Tf: transferrin
TGN: trans Golgi network
Vti1: Vps10 tail interacting 1
WT: wild‐type

## 1 Introduction

Transport between different organelles of the secretory and endosomal pathway requires membrane fission and membrane fusion in eukaryotic cells. Membrane fusion is mediated by specific members of the family of SNARE proteins on both membranes [1]. SNAREs are divided into Qa‐, Qb‐, Qc‐, and R‐SNARE subfamilies according to sequence similarities [2]. One SNARE motif from each subfamily combine to a four‐helix bundle in a specific fusion step. The Qb‐SNARE vti1a is found in the Golgi apparatus, the trans Golgi network (TGN), early endosomes and synaptic vesicles [3, 4]. Vti1a and vti1b share about 30% of their amino acid sequence and partially colocalize. However, vti1b also localizes to late endosomes and lysosomes. In in vitro assays, vti1a functions in the fusion of early endosomes and in retrograde transport to the TGN, while vti1b is required for fusion with late endosomes and lysosomes [5]. Only minor defects are observed in specialized cells in knockout mice lacking either vti1a or vti1b and the mice are viable and fertile. Cells of the adrenal medulla of vti1a deficient mice show defects in dense core granule biogenesis [6]. Lack of vti1b results in impaired secretion of lytic granules in cytotoxic T‐cells [7]. By contrast, vti1a*^−/−^* vti1b*^−/−^* double deficient (DKO) mice die at birth with major defects in the nervous system [8]. Some major axon tracts are missing, reduced in size or misrouted and several ganglia display neurodegeneration. Layer 5 in the cerebral cortex is malformed, the neural progenitor cell pool is depleted and apoptosis increased [9]. Neurite outgrowth is reduced, less synapses form and the amount of synaptic vesicle proteins is reduced in neuronal cell culture. The Golgi structure is altered and does not extend into dendrites in DKO neurons in cell culture and in vivo [8, 10, 11].

In order to investigate whether synaptic vesicle proteins are also reduced in vivo and to obtain unbiased insights into the molecular changes resulting from the absence of vti1a, vti1b or both of these SNAREs, we used shotgun proteomics from cerebral cortices of embryonic mice (E18.5) with these genotypes and compared them to wild‐type (WT) and double heterozygote (DHET) control samples.

## 2 Material and Methods

### 2.1 Animals

Generation and first characterization of mouse strains have been described: vti1b*^−/−^*, vti1a*^−/^*,*^−^* and vti1a*^−/−^* vti1b*^−/−^* [8, 12]. Animals were bred into the C57BL/6JRj mouse background (Janvier) in the mouse facility of Bielefeld University. Mice were sacrificed by CO_2_ gas inhalation and cervical dislocation in accordance with relevant guidelines and regulations and this was reported to local administrative office as required by German law. The cortices from individual embryos were isolated on embryonic development day 18.5 (E18.5) after timed mating. Genotype was determined by polymerase chain reaction.

### 2.2 Sample Preparation, Proteolytic Digestion, and LC–MS/MS

Mouse brain cortices were isolated at embryonic day E18.5, meninges and blood vessels removed in ice cold PBS, placed on dry ice and stored at −80°C. Samples were thawed on ice, 200 µL 100 mM ammonium bicarbonate, 200 µL trifluoroethanol and 10 µL 200 mM dithiothreitol added and mixed. The tissue was homogenized with ceramic beads using a precellys 24 (Peqlab) 3 times for 15 s at 5000 rpm and incubated for 1 h at 60°C. 200 µL 200 mM iodoacetamide was added before a 90 min incubation at room temperature. An additional 60 min incubation at room temperature followed after addition of 10 µL 200 mM dithiothreitol. 350 µL of this sample were removed and mixed with 437 µL 200 mM ammonium bicarbonate and 874 µL water [13, 14, 15].

**FIGURE 1:**
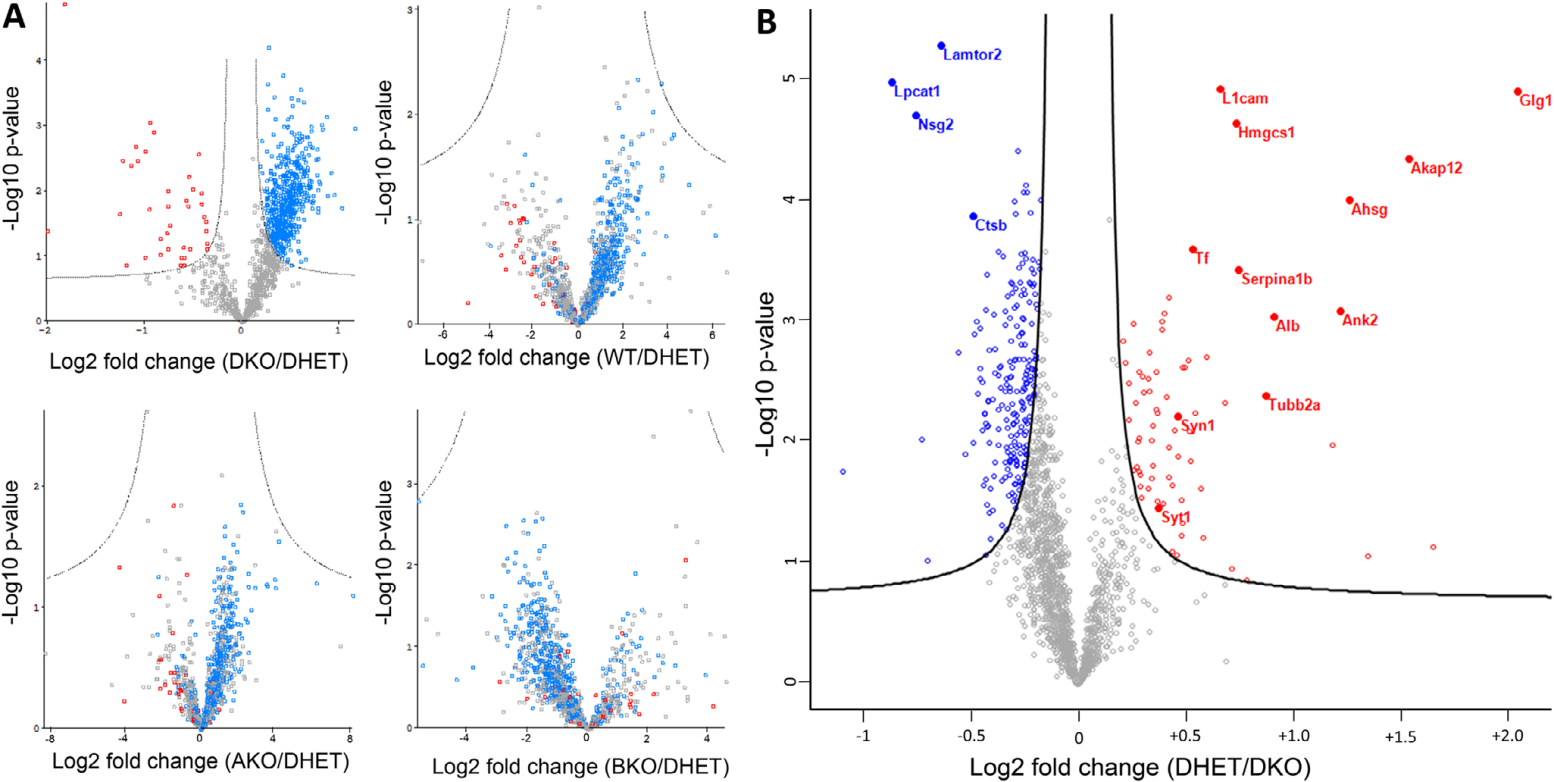
Hawaii and volcano plots of proteome analysis of E18.5 cerebral cortices. (A) Hawaii plot of the initial datasets, consisting of *n* = 5 (DHET (vti1a *^+/−^* vti1b*^+/−^* DHET), WT, AKO (vti1a*^−/−^* knockout), BKO (vti1b*^−/−^* knockout)), and *n* = 3 cortices (DKO (vti1a*^−/−^* vti1b*^−/−^* double knockout)). Each volcano plot displays the results of proteomic comparisons in log2 transformed changes in protein abundance between the mean abundance in the DHET group and one of the other genotype groups. –Log10 transformed *p*‐values are plotted. Significant differences were determined using a permutation‐based false discovery rate. The lines represent the thresholds using the parameters FDR = 0.01 and *s*_0_ = 0.1. Only the DHET/DKO plot displayed significant differences in protein abundance, which are colored blue for proteins with higher abundance in DKO embryos and red for less abundant proteins. No significantly changed proteins were identified in DHET/WT, DHET/AKO, and DHET/BKO sets. The proteins, which differed in abundance between DHET and DKO are colored blue or red accordingly to show their positions in the other plots but their abundance did not differ significantly. (B) Volcano plot based on an independent core set of *n* = 5 (DHET and DKO) E18.5 cortices and 1725 proteins that were identified in at least three biological replicates and quantified. Log2 transformed changes in protein abundance between both genotypes are plotted against –log10 transformed *p*‐values. Proteins that are significantly (FDR = 0.05) more abundant in DKO cortices compared to DHET cortices are colored blue and those that are significantly less abundant in DKO are shown in red. Highly significant changes (FDR = 0.01) as well as the synaptic vesicle proteins synaptotagmin 1 and synapsin 1 are highlighted as filled circles with gene names.

Protein concentrations were determined by DC assay (BioRad) and 100 µg protein was digested by 1 µg trypsin (trypsin gold, Promega) at 37°C overnight. Digested peptides were purified using SepPak columns (Waters, Milford, United States) and quantified with a nanodrop 2000 (Peqlab, VWR, Radnor, Pennsylvania, United States). Peptides were analyzed using a nanoLC (Ultimate 300, Thermo Fisher Scientific, Waltham, MA, US) coupled to a modified ESI‐Orbitrap MS/MS (QExactive Plus, Thermo Fisher Scientific, Waltham, MA, US) equipped with an enrichment and analytical column (Acclaim PepMap 100 C18: 0.3 mm diameter, 5 mm length, 5 µm particle size; Acclaim PepMap 100 C18: 0.075 mm diameter, 250 mm length, 5 µm particle size). In this setup, the mass spectrometer is equipped with a Spectroglyph source (Spectroglyph, LLC, WA, US) including an ion funnel instead of an S‐lense [16]. 1 µg of peptides of each sample was loaded onto the enrichment column before switching in‐line with the analytical column at a flow rate of 300 µL/min. The gradient length of the analytical column was adjusted to 67 min (WT, DHET, DKO, AKO, and BKO; Figure 1A) or 187 min (DHET and DKO; Figure 1B) and 5% to 41% acetonitrile (buffer A: 2% acetonitrile and 0.1% trifluoroacetic acid; buffer B: 80% acetonitrile and 0.1% trifluoroacetic; gradient 4% to 50% B). Two cohort experiments including five replicates of each genotype (WT, DHET, DKO, AKO, and BKO for the first experiment; DHET and DKO for the second experiment) were analyzed in random order. Only three biological DKO replicates were included in the first analysis, because two additional DKO cortices did not yield enough peptides. The mass spectrometer was operated in data‐dependent top 10 acquisition mode using a resolution of 70,000 (AGC target of 3e6 and 64 ms maximum IT) in full MS mode. For the dd‐MS^2^, a resolution of 17,500, AGC target of 2e5 and a maximum IT of 100 ms was used. The spray voltage of the ESI source was set to 1.8 kV.

### 2.3 Identification of Proteins

MaxQuant software (2.4.11.0) was used for database search against the UniProt *Mus musculu*s (strain C57BL/6J) reviewed database [17, 18]. For protein identification in MaxQuant, the enzyme specificity was set to trypsin with a maximum number of two missed cleavages, as well as standard instrument settings for orbitrap‐based data. Methionine oxidation and N‐terminal acetylation were set as variable modifications. Fixed modifications were set for the carbamidomethylation of cysteine. Label‐free quantification was set to LFQ with fast LFQ enabled. Only unique peptides were used for quantification. The false discovery rate (FDR) for PSMs and proteins was set to 5%, whereas the FDR for site decoy fractions was used at the default of 1%. The decoy mode for FDR calculation was set to reverse. All other settings were set to default.

The statistical analysis of the MaxQuant database search was performed in Perseus [19] software version 1.6.10.43 or 1.6.13.0. Proteins identified by MaxQuant as “only identified by site”, “reverse”, or “potential contamination” were removed. The ratio between the mean amounts of a protein in both genotypes was log2 transformed for visualization of differential protein abundance. Proteins with less than 2 identified peptides were filtered out. Additionally, only proteins were considered, which were detected in a minimum of three cortices out of the five cortices analyzed for a given genotype. The mass spectrometry proteomics data have been deposited to the ProteomeXchange consortium [20] via the PRIDE [21] partner repository with the dataset identifier PXD066845.

### 2.4 Cortex Tissue Lysates and Western Blot Analysis

DHET and DKO cortex lysates were prepared by homogenizing and lysing in RIPA buffer (25 mM Tris‐HCl pH7.6, 150 mM NaCl, 1% (v/v) NP‐40, 1% sodium desoxycholate, 0.1% SDS). Protein concentrations were determined by DC assay (BioRad) based on the manufacturer´s protocol. The samples were thermally denatured using an SDS containing buffer (final concentration 10% sucrose, 3.3% SDS, 135 mM Tris‐HCl pH 6.8, 3.3% β‐mercaptoethanol). The lysates (15, 20, or 30 µg) were loaded onto a 12.5% SDS‐PAGE gel. Proteins were electrophoretically transferred to nitrocellulose membranes by wet blot in 19 mM Tris HCl, 190 mM glycin pH 8.3, 20% methanol with 400 mA for 2 h. The absence of stained markers on the gel was assessed to monitor transfer efficiency. Ponceau stainings were performed for quality control and an image taken with a FujiFilm LAS‐3000 Luminescent image analyzer for quantification of the whole lane and normalization. Blots were cut to allow for normalization to GAPDH signal. Membranes were blocked in TBST blocking solution (150 mM NaCl, 25 mM Tris‐HCl pH 7.4, and 0.1% (v/v) Tween) with 5% (w/v) milk powder for 30–60 min followed by primary antibody incubation in TBST with 5% bovine serum albumin with one of the following antibodies: GAPDH (D16H11, Cell Signaling, 1:1000), Lpcat1 (16112‐1‐AP, Proteintech, 1:750), Nsg2 (ab189513, abcam, 1:1000), synaptobrevin (cl 69.1, Synaptic Systems; 1:1000), synaptophysin (cl 7.2, Synaptic System, 1:10), synaptotagmin (cl 41.1, Synaptic Systems, 1:1000), and SV2 (DSHB, 1:1000) overnight at 4°C. Blots were washed three times with TBST for 10 min, incubated with secondary antibodies (goat anti‐mouse‐HRP or goat anti‐rabbit‐HRP, 1:10000 in TBST with 2% milk powder) for 1 h at room temperature and washed three times with TBST for 10 min. Protein bands were visualized with WesternBright Chemiluminescence Kit (Advansta) in a FujiFilm LAS‐3000 Luminescent image analyzer. The quantification of the digital images was performed with ImageJ and the PlugIn Gel Analyzer without background correction.

### 2.5 Statistics

For analyzing the proteome data set with five different genotypes (DHET, WT, DKO, AKO, and BKO) Perseus (version 1.6.10.43) was used to generate the Hawaii plot and the principal component analysis (PCA) plot [19]. The Hawaii plot (multi‐volcano analysis) was created with a standard setting in Perseus as described [22]. Significant differences were determined using a permutation‐based false discovery rate. It is possible to define two different confidence classes with different FDR thresholds, class A (higher) and class B (lower confidence) in Perseus. The parameters FDR = 0.01 and *s*_0_ = 0.1 were chosen globally to obtain class A thresholds. *s*_0_ represents the background variance parameter in a modified t test statistic first introduced in the significance analysis of microarrays (SAM) method [23]. To determine the differences between DHET and DKO cortices Perseus (version 1.6.15) was utilized to create the volcano plot and the heatmap as well as the PCA analysis.

Quantification of the digital images of Western blots were performed with ImageJ (version 1.53t National Institute of Health). Statistical significance was calculated using Prism7 (GraphPad) and data sets were analyzed by student´s *t*‐test. *p*‐values < 0.05 were considered significant.

## 3 Results

### 3.1 Overview of the Protein Quantification in Different Vti1a and Vti1b Genotypes

**FIGURE 2:**
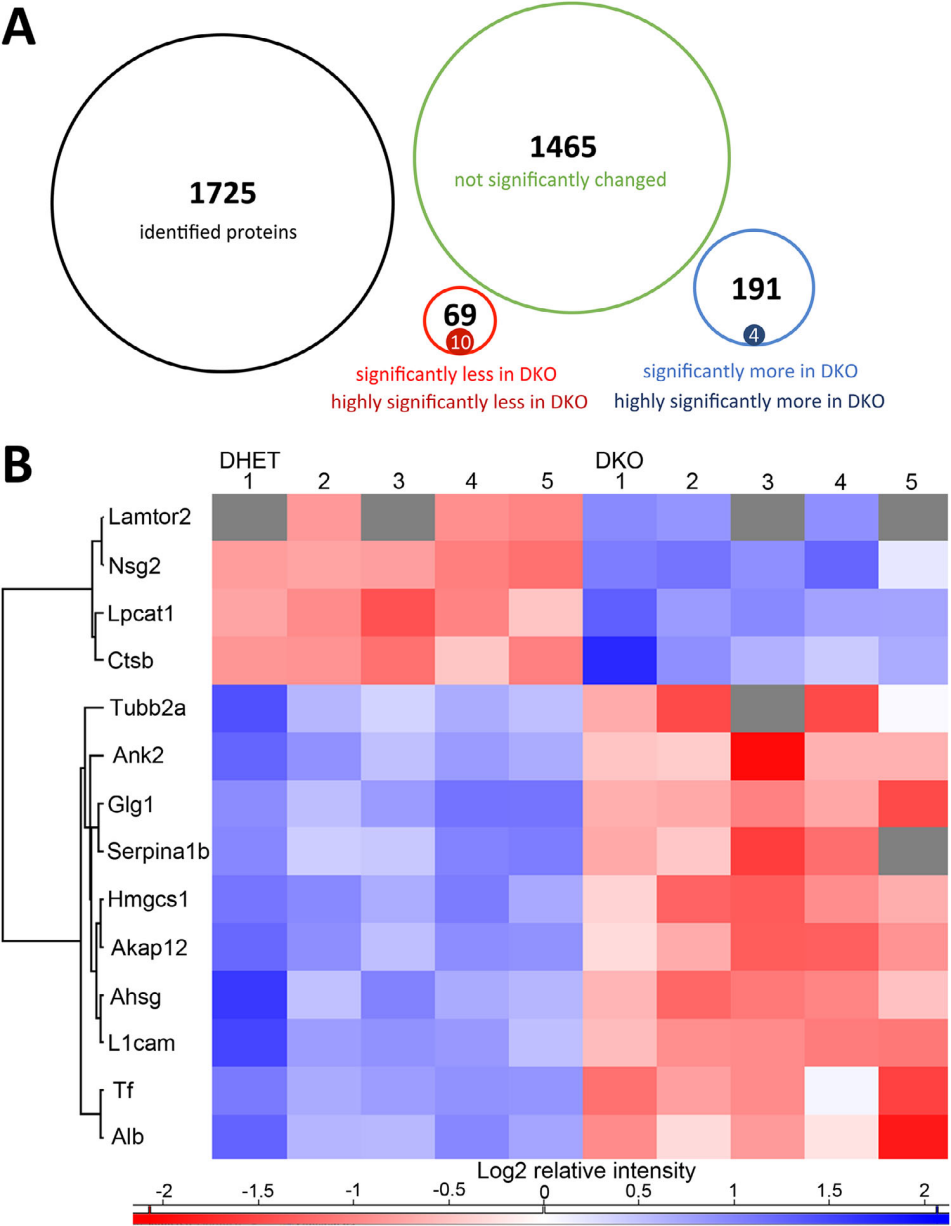
Proteins with differential abundance between DHET and DKO cortices. (A) Diagram of the 1725 identified proteins in DHET and DKO E18.5 cortices (black circle). 1465 Proteins were not significantly changed in their relative abundance (green circle). 69 proteins (red circle) were significantly less abundant in DKO cortices and 191 proteins (blue circle) were significantly more abundant (FDR = 0.05). Highly significantly (FDR = 0.01) more (4) or less (10) abundant proteins are shown in the smaller filled circles. (B) Heat map with hierarchical clustering of the 14 highly significantly more or less abundant proteins in each analyzed cortex. Blue shows a higher log2 relative intensity and red a lower log2 relative intensity compared to the average amount of each protein in cortices DHET1–DHET5 and DKO1–DKO5. Grey: protein is not detected in the sample.

Vti1a*^−/−^* vti1b*^−/−^* (DKO) embryos are not viable after birth and therefore embryonic cerebral cortices were used [8]. Mouse cerebral cortices were obtained from E18.5 embryos for proteomic analysis to allow for maximal time for development of the embryonic brain. Cortices of DKO, vti1a*^+/−^* vti1b*^+/−^* (DHET), WT, and vti1a*^−/−^* (AKO) and vti1b*^−/−^* (BKO) embryos were used for a first “shotgun MS” analysis to gain an understanding of the proteomic differences of all genotypes. Three biological DKO replicates and five biological replicates for all other genotypes were included in the analysis, because two additional DKO cortices did not yield enough peptides during mass spectrometry due to sample preparation. Embryonic cortices from the same litters were prepared except for WT, which could not be obtained due to the breeding schema. Protein extraction was followed by protein quantification, a tryptic digest and a MS sample preparation workflow. A label‐free quantification (LFQ) was executed in MaxQuant to identify differences between genotypes [17, 18]. Statistical proteomics data analysis was performed through Perseus software [19]. A principal component analysis (PCA) of relative protein abundance in the 23 samples revealed that the DKO samples clustered separately (Figure S1). There was no clear separation between samples derived from WT, DHET, AKO, and BKO cortices. The obtained Hawaii plot visualizes the results of *t*‐tests between DHET‐datasets with the indicated other genotypes in form of multiple volcano plots with a permutation‐based false discovery rate (FDR) of lower than 0.01 and *s*_0_ = 0.1 [22]. A total of 1209 proteins were identified in all three DKO and at least three individuals of each other genotype group in the first MS analyses. A total of 638 proteins showed significantly different abundances in DKO compared to DHET datasets (colored blue for proteins with higher abundance in DKO embryos and red for less abundant proteins; Figure 1A, top left). There were no significant differences in the protein abundance levels between DHET and WT, AKO, and BKO, respectively (Figure 1A). The proteins, which differed in abundance between DHET and DKO were also colored blue or red accordingly to show their positions in the other plots but their abundance did not differ significantly. A second shotgun MS analysis was performed with five DHET and five DKO cortices to further validate the protein differences. Again, a PCA showed that both genotypes were clearly different (Figure S2). A total of 1725 proteins were identified in at least three biological replicates of DHET and DKO (Table S3) and the change in abundance visualized in a volcano plot (Figure 1B). By grouping of replicates and performing a two‐sample *t*‐test (permutation‐based FDR < 0.05), a list of 260 proteins differed significantly in protein levels between DKO and DHET cortices. Detailed quantitative information is available at the online PRIDE database via ProteomeXchange with identifier PXD066845 [20, 21]. 191 proteins were significantly more abundant in DKOs and 69 proteins significantly less abundant in comparison with DHETs (Tables S4 and S5; Figure 2A). There were 14 highly significant changes in protein levels identified (FDR < 0.01). 4 proteins were more and 10 were less often detected in DKO compared to DHET cortices. A heatmap with relative quantitative log2 ratios of the individual biological replicates showed that these differences were reproducible (Figure 2B). However, some proteins were not detected in all samples (Figure 2B, grey squares). Taken together no differences in proteomes of WT, AKO, and BKO in comparison with DHET could be observed. DHET and DKO cortices showed strong differences on proteome level in a multiplicity of cellular processes.

### 3.2 DKO Cortex Proteomes Showed Higher Abundance of Nsg2, Lpcat1, and Proteins Connected to Ribonucleoprotein Complexes and Energy Metabolism

**FIGURE 3:**
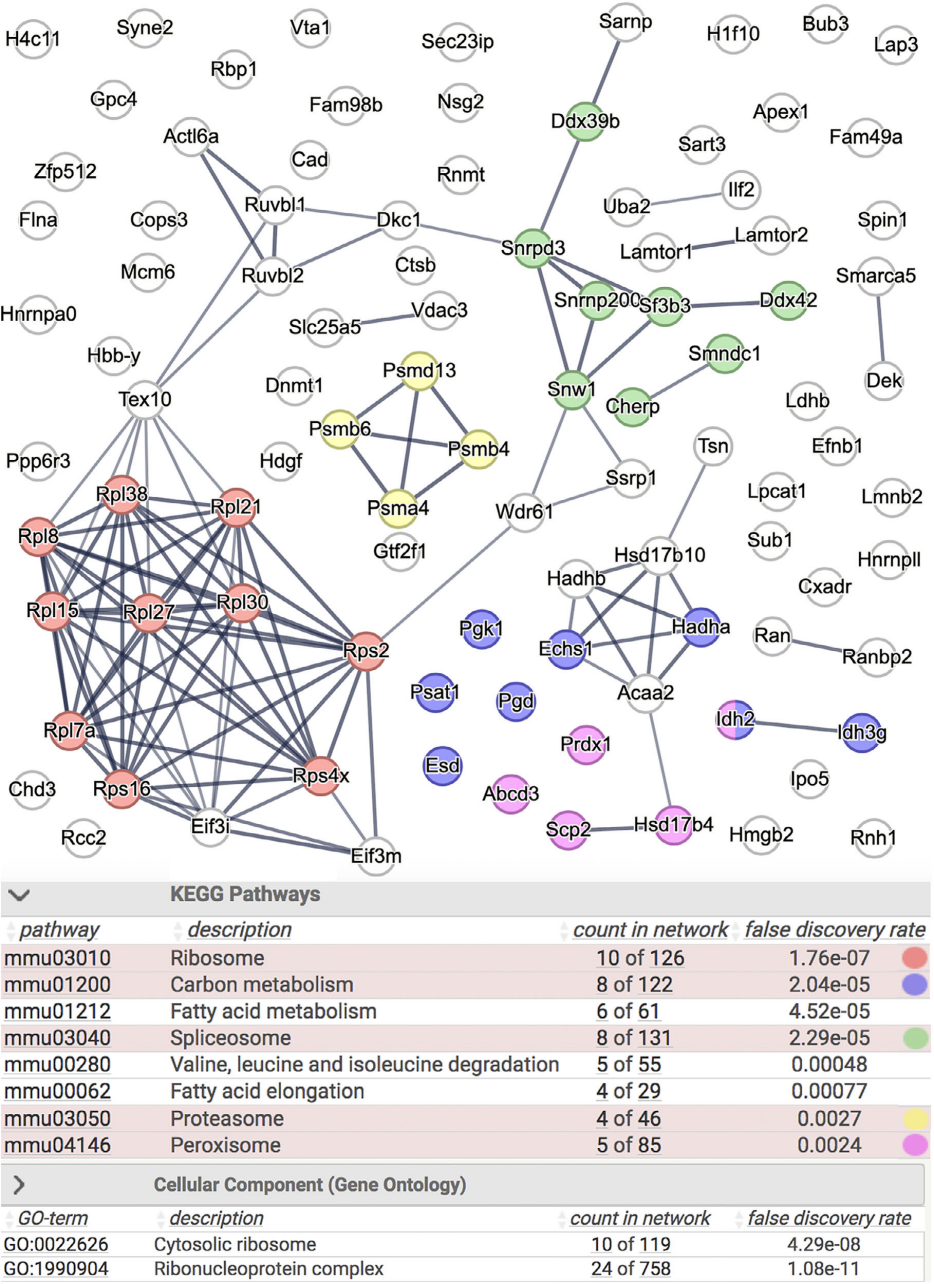
Pathway enrichment for the 98 proteins significantly more abundant in DKO cerebral cortices with a FDR = 0.03. Top: STRING functional protein association network. The FDR = 0.03 was used to reduce the number of proteins for better visualization. Bottom: terms highly enriched in KEGG‐pathways and GO cellular components. The same terms appeared at the top of the lists using a FDR of 0.03 (98 proteins) and 0.05 (191 proteins). The thickness of connecting lines indicates the strength of data support for an association.

A STRING functional protein association network [24] was created with all 191 proteins with higher abundance in the DKO cortex proteome (data not shown) and with the 98 proteins more abundant with a FDR < 0.03 (Figure 3, top) to obtain insights into the affected biological processes and pathways. Networks were identified with Kyoto Encyclopedia of Genes and Genomes (KEGG) pathways and gene ontology (GO) databases and enrichment analysis performed to identify proteins, which were overrepresented among the more abundant proteins compared to the total number of proteins in the network (Figure 3, bottom). Both analyses gave similar results. Proteins connected to ribosomes, spliceosomes, and ribonucleoprotein complexes were highly overrepresented. Pathways such as carbon metabolism, fatty acid degradation, and the degradation of amino acids such as valine, leucine, and isoleucine were also enriched. All are relevant for an intact energy metabolism of eukaryotic cells and point toward an alteration in DKO cortices. Lamtor2, cathepsin B, Lpcat1, and Nsg2 were proteins that were present in particularly high amounts in DKO cortices. Lamtor2 is a subunit of the ragulator complex, which recruits mTORC1 to lysosomes and plays a role in nutrient sensing in cell growth and metabolism [25]. Cathepsin B is a lysosomal protease [26]. However, lysosomes, endosomes, autophagosomes, Golgi or vesicles connected with these organelles are not among the cellular components listed as functional enrichment. Lpcat1 is short for lysophosphatidylcholine acyltransferase and uses saturated fatty acids to create the phospholipid phosphatidylcholine [27, 28]. Nsg2 regulates the endosomal recycling of AMPA receptors in neurons [29].

### 3.3 DKO Cortex Proteomes Showed Lower Abundance of Proteins Involved in Synaptic Vesicles and Synapses

**FIGURE 4:**
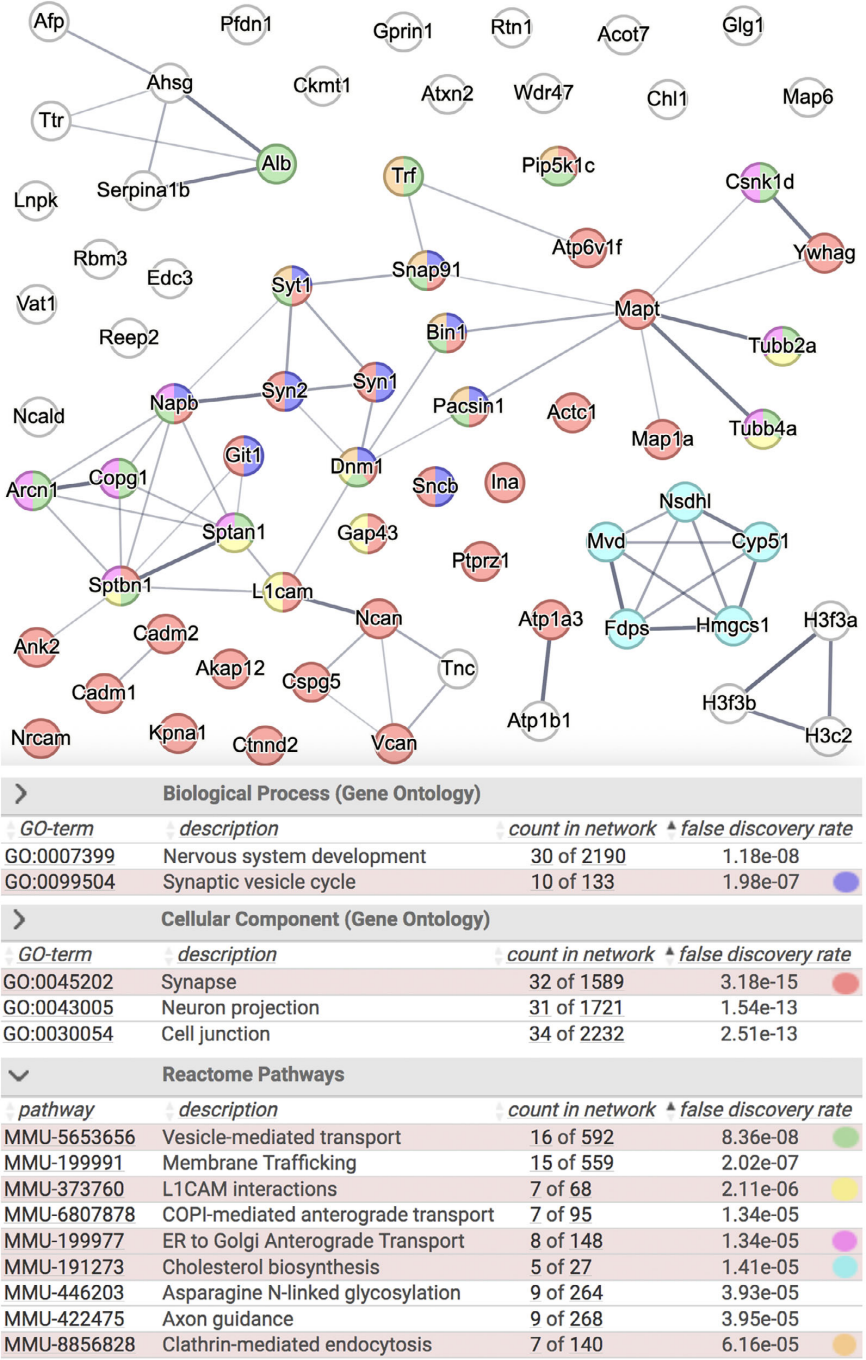
Pathway enrichment for the 69 proteins significantly less abundant in DKO cerebral cortices. Top: STRING functional protein association network. Bottom: terms highly enriched in GO and reactome pathways. The thickness of connecting lines indicates the strength of data support for an association.

A STRING functional protein association network was created with all 69 proteins with lower abundance in the DKO cortex proteome (Figure 4). Almost half of these proteins were connected with the GO term synapse. Among them were 10 proteins involved in the synaptic vesicle cycle including the synaptic vesicle proteins synaptotagmin 1, synapsin 1, and synapsin 2 and seven proteins connected to clathrin mediated endocytosis. Also encompassed under the GO term synapse were the cell adhesion molecules L1cam, Cadm1, Nrcam, and Chl1 as well as the proteoglycane core proteins Vcan, Ncan (Cspg2), and Cspg5 as their potential interaction partners. L1cam was highly significantly reduced in DKO cortices. Several proteins belonged to the reactome pathways L1cam interaction and axonal guidance including the highly significantly reduced tubulin‐ß‐2A chain encoded by the Tubb2a gene. Ankyrin‐2 (Ank2) interacts also with L1cam [30, 31] and was highly significantly reduced in DKO cortices. Other enriched reactome pathways were cholesterol biosynthesis and the overlapping terms vesicle‐mediated transport, membrane trafficking, ER to Golgi anterograde transport and COP1‐mediated anterograde transport. Four highly significantly reduced proteins were constituents of the blood serum: albumin (Alb), fetuin (Ahsg), transferrin (Tf), and α1‐antitrypsin 1–2 (serpin1b) [32].

### 3.4 Verification by Western blot

**FIGURE 5:**
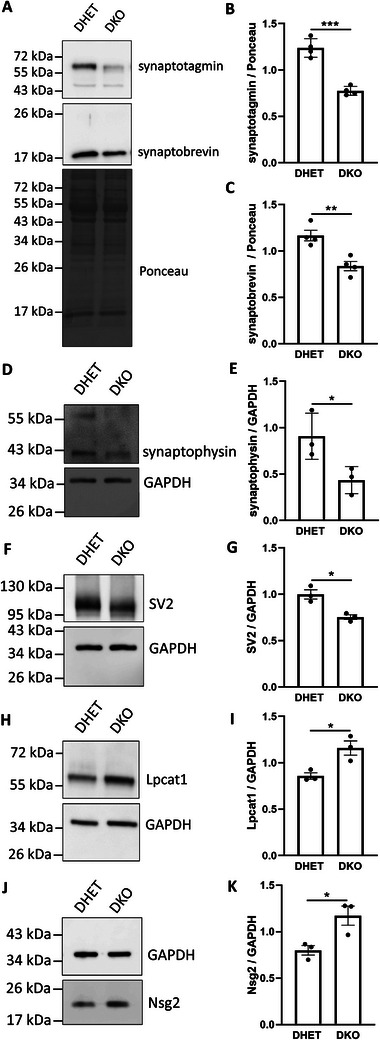
Western blot analysis of DHET and DKO cerebral cortices. (A–G) Protein lysates (30 µg) from cortices of E18.5 DHET and DKO embryos were analyzed by Western blot for synaptobrevin (18 kDa), synaptophysin (38 kDa), synaptotagmin 1 (60 kDa), and SV2 (95 kDa). Analyses revealed significant decreases of synaptic vesicle markers in DKO cortices. (H–K) For verification of Lpcat1 (59 kDa) 15 µg protein and for detection of Nsg2 (19 kDa) 20 µg protein were analyzed by Western blots. An increase of both proteins could be determined in DKO cortices compared to DHET. For quantification of the protein ratio Ponceau staining or GAPDH (35 kDa) was used as internal loading control (synaptophysin, SV2, Lpcat1, Nsg2: *n* = 3 cortices, synaptobrevin, and synaptotagmin: *n* = 4 cortices of each genotype, the mean of two Western blot replicates for each cortex is depicted for synaptobrevin and Lpcat1). Bars are the mean ± SD (B, E, G, and K) or SEM (C and I); *: *p* < 0.05; **: *p *< 0.01; ***: *p* < 0.0001; unpaired *t*‐test.

Next, we wanted to verify and extend the mass spectrometry data by Western blot analysis focusing on synaptic vesicle proteins. The synaptic vesicle integral membrane proteins synaptobrevin, synaptophysin, and SV2 were not among the 1725 proteins detected by mass spectrometry. A significant difference in protein abundance between DHET and the single knockout brains could not be shown based on the LC–MS/MS‐data. Therefore, we performed Western blot only on DHET and DKO lysates. The amount of the synaptic vesicle proteins synaptophysin, synaptobrevin, synaptotagmin 1, and SV2 were significantly decreased by 25% to 50% in DKO cortices (Figure 5A–G) confirming the mass spectrometry data for synaptotagmin and identifying three additional synaptic vesicle membrane proteins with reduced abundance in DKO cortices. On the other hand, we took a closer look at two proteins, Lpcat1 and Nsg2, which were more abundant in the DKO cortex by LC–MS/MS. The Western blot indicated that Lpcat1 was significantly increased in DKO cortices compared to DHET (Figure 5H,I) as well as to vti1a*^−/−^* and vti1b*^−/−^* cortices (Figure S6). Nsg2 was also more abundant in DKO cortices (Figure 5J,K).

## 4 Discussion

In principle component analysis, DKO samples were clearly distinct from all other genotypes studied. By contrast, WT, DHET, vti1a*^−/−^* and vti1b*^−/−^* cerebral cortices were not clearly separated. These data reflect the observations in living animals: DHET are indistinguishable from WT mice, minor phenotypes are detected in vti1a*^−/−^* and vti1b*^−/−^* mice in specialized cells such as adrenal chromaffin cells and cytotoxic T‐cells while DKO mice die at birth [6, 7, 8]. 1725 proteins were identified in at least three of the five DHET and DKO cortices, which is typical for an analysis of this type [13]. However, this constitutes only a small fraction of proteins present in brain. In addition, this shotgun mass spectrometry approach identifies tryptic peptides and infers the presence of the full‐length protein. Many mRNAs undergo alternative splicing leading to different proteoforms, which cannot be detected by this method. Posttranslational modifications were not studied.

Among the 69 proteins, which were detected in reduced amounts in DKO cerebral cortices, 10 proteins were connected with the synaptic vesicle cycle including the synaptic vesicle proteins synaptotagmin, synapsin1, and synapsin 2. Immunoblots detecting the synaptic vesicle proteins synaptotagmin, synaptophysin, synaptobrevin, and SV2 showed that these four proteins were present in reduced amounts in DKO cerebral cortices. These data confirmed the mass spectrometry results for synaptotagmin. Synaptophysin, synaptobrevin, and SV2 were not among the 1725 proteins identified frequently enough by mass spectrometry. The immunoblot data showed that several synaptic vesicle proteins were reduced in DKO cortices in parallel indicating that synaptic vesicles may be less abundant in the DKO embryonic brain. A significant reduction of synaptotagmin and a trend toward a reduction of synaptobrevin has been observed in synapses of DKO hippocampal neurons cultivated for two weeks in cell culture by immunofluorescence [10]. These authors also observed a reduction in proteins required for synaptic vesicle exocytosis such as the plasma membrane Qbc‐SNARE SNAP‐25, the regulatory proteins Munc13‐1 and RIM1/2 as well as the active zone scaffold protein Bassoon. They observed that the number of synapses per µm of neurite is reduced but similar numbers of synaptic vesicles per synapse were detected by electron microscopy in the absence of vti1a and vti1b in cell culture. Neurites were also shorter further reducing the number of synapses. Golgi export and retrograde transport to the Golgi is reduced in these cells. These defects could contribute to the observed reduction of synaptic proteins in cortex. In addition, synaptic vesicle secretion was reduced in cultured neurons [10]. Differentiated vti1a and vti1b deficient N1E‐115 neuroblastoma cells contain less synaptotagmin, synaptophysin, synaptobrevin, and SV2 than differentiated WT N1E‐115 cells [33].

Five proteins connected with the gene‐ontology biological process “synaptic vesicle cycle” were also part of the reactome pathway “clathrin‐mediated endocytosis”, namely dynamin 1 (Dnm1), amphiphysin 1 (Bin1), pacsin1 (syndapin), AP180 (SNAP91), and ß‐SNAP (Sncb). ß‐SNAP recruits NSF for dissociation of SNARE‐complexes while AP180 is an endocytic adaptor for the SNARE synaptobrevin [34]. Dynamin, amphiphysin, and pacsin remodel membranes in clathrin‐mediated endocytosis [35]. The phosphoinositide kinase Pip5k1c was also reduced in abundance and is connected to the reactome pathway “clathrin‐mediated endocytosis” because clathrin coated vesicles form at PI(4,5)P_2_ rich plasma membrane patches [36]. Therefore it can be speculated, that recycling of synaptic vesicles after exocytosis and other clathrin‐mediated endocytosis pathways may be affected.

Protein levels of the cell adhesion molecule L1cam and its interaction partner ankryin‐2 (Ank2) [31] were highly significantly reduced. Knock out mice deficient for L1cam or Ank2 display hypoplasia in several axon tracts and Ank2 deficient axons contain less L1cam emphasizing their role in neurites [30]. DKO embryonic brains also display defective long axon tracts [8]. L1cam has to be recycled through endosomes in axonal growth [37]. Reduced amounts of L1cam could be caused by a defective endosomal system in DKO brains and contribute to the observed phenotypes. On the other hand, lowered L1cam levels could be a consequence of the reduced neurite development in DKO brains. Interestingly, additional cell adhesion molecules (Cadm1, Nrcam, and Chl1) were less abundant as well as several proteoglycan core proteins (Vcan, Ncan, and Cspg5) as their potential interaction partners.

Among the 10 highly significantly reduced proteins in DKO cortices are four proteins, which are found in the blood serum: albumin (Alb), fetuin (Ahsg), transferrin (Tf), and α1‐antitrypsin 1–2 (serpin1b). By contrast, the α and β chains of hemoglobin were detected in equal amounts in DKO and DHET cortices indicating that similar amounts of red blood cells were present in brain. Therefore, a specific reduction of blood serum proteins was observed. One possible explanation is that biosynthesis and constitutive secretion of Alb, Ahsg, Tf, and serpin1b is affected, which occurs mainly in the liver for these proteins [32]. The formation of secretory granules and their regulated secretion is affected in the absence of vti1a and vti1b [6, 10], which may be due to membrane removal during maturation of secretory granules and problems in the endosomal system. Therefore, it is possible that constitutive secretion is also affected because their membranes also recycle through endosomes after exocytosis. However, constitutive secretion is the default pathway leaving the TGN, does not require vesicle maturation and may be less sensitive to the loss of vti1a and vti1b. On the other hand, transferrin crosses the blood brain barrier by transcytosis with endocytosis as first step followed by exocytosis [38]. The blood brain barrier differentiates between E15 and E18 and increases in tightness during this time [39]. Fetuin is also transported from the serum to the cerebrospinal fluid [40]. Transcytosis through the lung endothelium has been reported for serpin1 [41]. Therefore, defects in the endosomal system could affect transcytosis and could be responsible for the reduced amounts of these serum proteins in DKO cortices. For Alb, a mechanism has been reported, which increases its lifespan. Alb is constitutively endocytosed, but can be recycled by binding to the neonatal Fc receptor for IgG (FcRn) in endosomes [42]. Plasma Alb concentration is reduced by 50% in FcRn deficient mice because it is not recycled but degraded in lysosomes. The absence of the Golgi and endosomal SNAREs vti1a and vti1b could reduce this recycling and transcytosis leading to the reduced levels of these proteins observed in DKO cortices. Proteins connected to vesicles in the network were enriched with a FDR lower than 1e‐05. The cellular component Golgi apparatus appeared with an FDR of 0.0036 and endosome, lysosome or autophagosome were not listed. Five enzymes of the cholesterol biosynthesis pathway were less abundant. ER to Golgi traffic of a transcription factor regulates transcription of the rate‐limiting enzyme HMG‐CoA reductase [43], but it was not among the detected proteins. Future study could analyze cholesterol concentrations.

With our shotgun approach, 191 proteins could be also detected, which were significantly increased in DKO cortices. These more abundant proteins were analyzed according to the pathways they participate in. As most notable pathways they belong to spliceosomes, ribosomes and carbon metabolism. These are basic cellular pathways, which could vary in abundance dependent on the cell type. It has been observed that the cortical progenitor cells are depleted faster in DKO cortices, more apoptosis was detected and the cortical layer 5 was disorganized [9]. Therefore, there is a shift in fractions of different cell types in DKO compared to DHET cerebral cortices, which could cause the increased detection of proteins in mRNA processing and protein biosynthesis. Alternatively, the reduction in normally plentiful synaptic proteins and the loss of axon tracts observed in brain sections [8] may increase the relative abundance of other proteins noticeably in DKO cortices. The synaptic vesicle proteins synaptophysin and synapsin are enriched about 20‐fold in highly purified synaptic vesicles compared to adult mouse brain homogenate indicating that all synaptic vesicle proteins together account for about 5% of total brain protein [44].

Two highly significantly increased proteins were validated by immunoblotting of DKO brain extracts, lysophosphatidylcholine acyltransferase 1 (Lpcat1) and neuron‐specific gene 2 (Nsg2). Nsg2 binds to AMPA receptors in postsynaptic densities and is involved in trafficking of AMPARs between the plasma membrane and endosomes [29]. Nsg2 is a short‐lived protein due to degradation in lysosomes and can be stabilized by overexpression of a dominant negative variant of the late endosomal rab7, which inhibits transport of Nsg2 to lysosomes [45]. Therefore, an impaired transport of Nsg2 from endosomes to lysosomes in the absence of vti1a and vti1b could explain the increased amounts of Nsg2 in DKO cortices.

Lpcat1 is an ER protein, which catalyzes the remodeling of phosphatidylcholine by transferring an acyl moiety. In lungs, this enzyme is important for synthesis of the surfactant component dipalmitoylphosphatidylcholine but it is also expressed in the brain [46]. Recently, upregulation of Lpcat1 was observed in two proteome studies involving neurodegeneration. Overexpression of an α‐synuclein variant connected with Parkinson's disease in cortical neurons resulted in increased amounts of Lpcat1 [47]. Lpcat1 was also more abundant in postmortem brain tissue from Parkinson's disease patients [47]. Lpcat1 amounts were increased in the striatum of mice without WD repeat domain 45 (WD45) in dopaminergic neurons before dopaminergic axonal degeneration in the striatum and death of dopaminergic neurons [48]. Upregulation of Lpcat1 may change the lipid composition and contribute to the neuronal pathology, which should be studied in the future.

Rab GTPases regulate membrane traffic. Rab1b involved in ER to Golgi transport and Rab11 on recycling endosomes were more abundant in DKO cortices. Several other members of the Rab GTPase family were also detected but not significantly changed in their amounts (Rab1a, Rab2a/2b), Rab3A, Rab5a, Rab5b, Rab5c, Rab7a, Rab14, Rab18, and Rab35).

In summary, our proteomic approach proves that only the loss of both vti1a and vti1b leads to major changes in the protein composition in the murine cortex. On the other hand, our data provide new insights into the molecular defects caused by the absence of Golgi, TGN and endosomal SNAREs vti1a and vti1b centered on the synapse and cell adhesion. Changed abundances in enzymes involved in the synthesis of three different lipids were unexpected. Lipid composition should be studied in the future, especially concerning phosphatidylcholine, cholesterol and phosphoinositides.

## Funding

The authors have nothing to report.

## Conflicts of Interest

The authors declare no conflicts of interest.

## Supporting information




**Supporting File 1**: pmic70117‐sup‐0001‐figures.pdf.

**Supporting File 2**: pmic70117‐sup‐0002‐tables.zip.

## Data Availability

The mass spectrometry proteomics data have been deposited to the ProteomeXchange consortium via the PRIDE partner repository with the dataset identifier PXD066845.
