# Supplementary material for: Reduction in Synaptic Vesicle Protein Abundance but Increased Amounts of Nsg2 and Lpcat1 in Cerebral Cortices Without the Endosomal SNARE Proteins Vti1a and Vti1b
Source: Proteomics. 2026 Mar 20;26(5):86–97. doi: 10.1002/pmic.70117 (PMC13106906; doi:10.1002/pmic.70117)
Supplement: Supplementary file 1 — Supporting File 1: pmic70117‐sup‐0001‐figures.pdf. [file PMIC-26--s001.pdf]

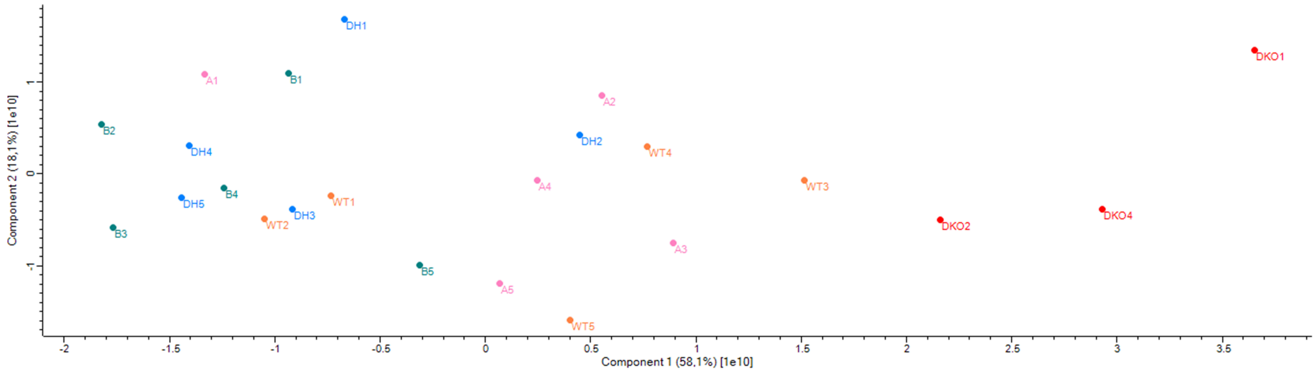

Fig. S1: Principal component analysis (PCA) plot of DHET, WT, vti1a and vti1b single and double knockout cortical samples.

PCA plot shows the variation between *Vti1a*$^{-/-}$ *Vti1b*$^{-/-}$ DKO cortices (n=3) and the other genotypes like WT, DHET (DH), AKO (A) and BKO (B) (n=5).

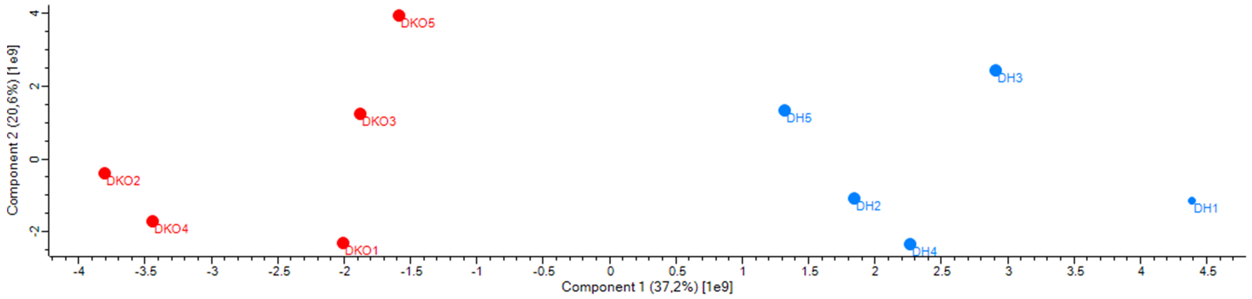

Fig. S2: Principal component analysis (PCA) plot of DHET and DKO cortical samples.

PCA plot shows the variation between DHET (DH) and *Vti1a*$^{-/-}$ *Vti1b*$^{-/-}$ DKO samples (n=5).

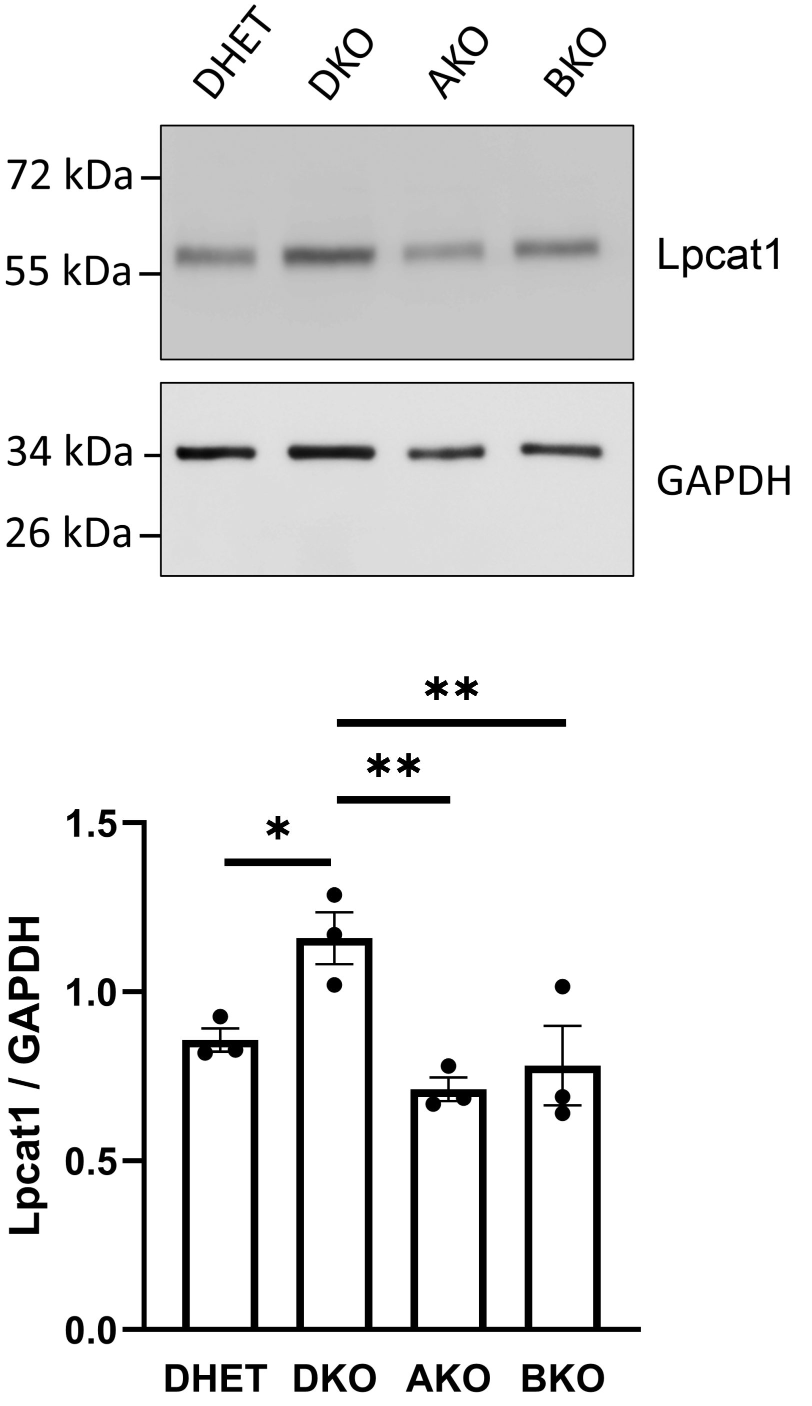

Fig. S6: Amounts of Lpcat1 were increased significantly in DKO compared to DHET, *Vti1a*$^{-/-}$ (AKO) and *Vti1b*$^{-/-}$ (BKO) cortices.

Protein lysates (15 µg) from cortices of E18.5 DHET and DKO embryos were analyzed by Western blot for Lpcat1 (59 kDa). GAPDH (35 kDa) was used as internal loading control for quantification. n = 3; Bars are the mean ± SEM; *: $p < 0.05$, **: $p < 0.01$, one-way ANOVA
